# Experience sampling self-reports of social media use have comparable predictive validity to digital trace measures

**DOI:** 10.1038/s41598-022-11510-3

**Published:** 2022-05-09

**Authors:** Tim Verbeij, J. Loes Pouwels, Ine Beyens, Patti M. Valkenburg

**Affiliations:** 1 Amsterdam School of Communication Research, University of Amsterdam, P.O. Box 15791, 1001 NG Amsterdam, The Netherlands; 2 Behavioural Science Institute, Radboud University, Nijmegen, The Netherlands

**Keywords:** Psychology, Human behaviour

## Abstract

Research agrees that self-reported measures of time spent with social media (TSM) show poor convergent validity, because they correlate modestly with equivalent objective digital trace measures. This experience sampling study among 159 adolescents (12,617 self-reports) extends this work by examining the *comparative predictive validity* of self-reported and digital trace measures of TSM, that is, the extent to which self-reported and digital trace measures of TSM correspond in their effects on self-esteem, well-being, and friendship closeness. Using an *N* = 1 method of analysis, we investigated the correspondence on a between-person, within-person, and person-specific level. Although our results confirmed the poor convergent validity of self-reported TSM reported earlier, we found that self-reports of TSM had comparable predictive validity to digital trace measures on all three levels. Because comparative predictive validity of self-reported TSM is crucial for investigating social media effects, our results have important implications for future research using self-reported TSM.

In the past decade, numerous studies have examined the associations of time spent on social media (TSM) with adolescents’ psychosocial functioning, broadly defined as adolescents’ ability to achieve their personal and social developmental goals^1^. Psychosocial functioning includes various developmental outcomes, most notably self-esteem, well-being, and friendship closeness^2–4^. Meta-analyses on the associations of TSM with psychosocial functioning have yielded diverging pooled effect sizes that varied across outcomes. Specifically, whereas the reported pooled associations of TSM with self-esteem and well-being were weakly negative, those with friendship closeness were moderately positive (self-esteem: *r* = − 0.09^5^; well-being: *r* = − 0.06 and − 0.07^6,7^; friendship closeness: *r* = 0.27^8^).

Most prior studies have used self-reported measures of TSM obtained via retrospective surveys or experience sampling methods (ESM)^9,10^. However, a recent meta-analysis revealed that self-reported measures typically correlate only modestly with digital trace measures of TSM^11^, so that their convergent validity has become a serious reason for concern. Yet, although self-reported measures of TSM show only modest convergent validity, they might still show comparative predictive validity to digital trace measures. That is, even though self-reported and digital trace measures only correspond weakly with each other, their effects on psychosocial functioning might be comparable. By comparing the predictive validity of self-reported and digital trace measures, Sewall et al.^12^ and Johannes et al.^13^ found that the between- and within-person associations between TSM and well-being yielded the same sign for self-reported and digital trace measures, although the associations were somewhat stronger for self-reported measures than for digital trace measures.

Although our understanding of the effects of TSM on psychosocial functioning has improved considerably in recent years, the literature leaves two important gaps that this study seeks to fill. A first gap in the literature is that we do not yet know whether the predictive validity of ESM self-reports and digital trace measures of TSM is comparable. Except for the studies by Sewall et al.^12^ and Johannes et al.^13^, hardly any study has focused on the *comparative predictive* validity of self-reported and digital trace measures of TSM by investigating to what extent self-reported and digital trace measures of TSM yield comparable effects on certain outcome variables. Given that Sewall et al.^12^ focused on retrospective surveys of TSM and that the ESM study by Johannes et al.^13^ only examined well-being as an indicator of psychosocial functioning, it is still unknown whether self-reported ESM estimates and digital trace estimates of TSM correspond in their prediction of different indicators of psychosocial functioning. Therefore, the first aim of the current study was to examine to what extent self-reported and digital trace measures of TSM have similar between-person and within-person associations with three components of psychosocial functioning: self-esteem, well-being, and friendship closeness.

A second gap in the literature is that it is yet unknown whether the comparative predictive validity of self-reported and digital trace measures of TSM differs across persons. Previous studies that adopted a person-specific (or *N* = *1*) paradigm, have shown that adolescents differ greatly in their susceptibility to the effects of TSM on self-esteem^14^, well-being^15^, and friendship closeness^16^. For example, one study showed that the effect of TSM on self-esteem is non-existent to very small for 56%, negative for 27%, and positive for 18% of adolescents^17^. Likewise, another study demonstrated that the effect of time spent browsing on well-being is non-existent to very small for 63%, negative for 20%, and positive for 17% of adolescents^18^. Yet, it is still unknown to what extent the person-specific effects of self-reported and digital trace measures correspond. For example, do adolescents who experience a negative effect on self-esteem according to a self-reported measure of TSM also experience a negative effect according to a digital trace measure of TSM? Therefore, the second aim of our study is to explore to what extent the person-specific effects of self-reported and digital trace measures of TSM on self-esteem, well-being, and friendship closeness correspond with each other and to what extent this correspondence differs across adolescents.

To address the two aims of the current study, we relied on a three-week experience sampling method (ESM) study among 159 adolescents. During this ESM study, adolescents reported six times per day how much time they had spent on three popular social media platforms (i.e., Instagram, Snapchat, and WhatsApp) and their momentary levels of self-esteem, well-being, and friendship closeness (on average 79 valid assessments per adolescent, 12,617 in total). In addition, we tracked adolescents’ time spent with these platforms across the three-week period using software designed to retrieve app usage from Android smartphones^19^. To assess the comparative predictive validity of self-reported and digital trace measures of TSM, we focused on three components of psychosocial functioning that have been investigated in relation to TSM: self-esteem (i.e., judgment of self-worth)^14,20^, affective well-being (i.e., feeling of happiness)^15,21^, and friendship closeness (i.e., supportive, responsive, and accessible peer relations)^16,22^. The current study is a follow-up study of Verbeij et al.^23^, which relied on the same dataset, but focused only on the convergent validity of retrospective survey and ESM measures of TSM.

To assess the comparative predictive validity of self-reported and digital trace measures of TSM, we used Dynamic Structural Equation Modeling (DSEM), a modeling technique that combines *N* = *1* time-series modeling, multilevel modeling, and structural equation modeling^24^. DSEM enabled us to assess the comparative predictive validity of self-reported and digital trace measures of TSM on three different levels: the between-person, within-person, and person-specific level. The *between-person* level analyses allowed us to investigate the correspondence in the between-person associations of self-reported and digital trace measures of TSM with psychosocial functioning (i.e., between-person comparative predictive validity). That is, whether adolescents who spend more time on social media than their peers have a higher (or lower) level of psychosocial functioning than their peers according to both the self-reported and digital trace measures.

The *within-person* analyses enabled us to investigate the correspondence in the longitudinal within-person effects of self-reported and digital trace measures of TSM on psychosocial functioning (controlled for previous levels of psychosocial functioning; within-person comparative predictive validity). That is, do adolescents who spend more time on social media than they usually do also have a higher (or lower) psychosocial functioning than they usually do according to both self-reported and digital trace measures? Finally, the *person-specific* or *N* = 1 component of DSEM allowed us to compute a person-specific effect of TSM on psychosocial functioning for each individual adolescent (i.e., an *N* = 1 beta). The 159 person-specific effect sizes were used to explore to what extent the effects of self-reported and digital trace measures of TSM on self-esteem, well-being, and friendship closeness correspond with each other within each adolescent and to what extent this correspondence differs across adolescents. Specifically, we examined the correlation between the person-specific effect sizes based on the self-reported and digital trace measure of TSM and explored whether adolescents who experienced a positive, negative, or null effect of self-reported TSM on psychosocial functioning experienced a comparable effect according to the digital trace measure of TSM (i.e., person-specific comparative predictive validity).

## Results

### Convergent validity of self-reports, accuracy of self-reports, and descriptive statistics

As Table 1 shows, the between-person correlation (*r* = 0.44) and the within-person correlation (*r* = 0.30) between self-reported and digital trace measures of TSM are indicative of poor convergent validity; they are below the threshold for minimally acceptable convergent validity of *r* = 0.50^11^. Comparably poor convergent validity has already been reported in an earlier study based on the same dataset^23^. As shown in this earlier study, adolescents tended to overestimate their TSM: On average, they spent about 14 min per hour on social media according to their self-reported TSM, but six minutes according to the digital trace measure. This difference was significant (*t*(158) = 9.37, *p* < 0.001, *d* = 0.74). The remaining descriptive statistics and the correlations among all study variables can be found in Table 1.

**Table 1:** Descriptive statistics and between-person, within-person, and intraclass correlations for all study variables.

	1	2	3	4	5
1. TSM—Self-reported	–	0.30***	− 0.04***	− 0.01	0.09***
2. TSM—Digital trace	0.44***	–	0.00	0.06***	0.08***
3. Self-esteem	− 0.23**	− 0.12	–	0.33***	0.23***
4. Well-being	− 0.28***	− 0.06	0.85***	–	0.20***
5. Friendship closeness	0.06	0.00	0.61***	0.55***	–
*M*	13.77	5.87	4.07	4.37	3.39
*SD*	15.68	9.37	1.43	1.44	1.79
Range	0–60	0–60	0–6	0–6	0–6
ICC	0.52	0.24	0.51	0.53	0.44

TSM = Time spent on social media. Mean scores for TSM reflect the average number of minutes spent of TSM in the hour previous to the ESM assessments. Correlations below the diagonal line represent between-person correlations, correlations above the diagonal line represent within-person correlations. For the correlations, estimates of TSM according to the self-reported and digital trace measures were log transformed. ICC = intraclass correlation.

*p < .05. **p < .01. ***p < .001.

### Comparative predictive validity of self-reported and digital trace measures

To address the two aims of our study, we investigated to what extent the self-reported and digital trace measures of TSM showed similar associations with self-esteem, well-being, and friendship closeness on the between-person, within-person, and person-specific level. The alpha levels of the reported *z*-tests (to compare the between-person associations) and *t*-tests (to compare the within-person effects) were adjusted to control for the number of tests we performed (α = 0.05/3 = 0.017). For the within-person effects, we also determined Cohen’s *d,* which reflects the magnitude of the difference in predictive validity between self-reported and digital trace measures of TSM. We used the following general guidelines: (a) *d* between 0 and 0.49 indicates that the predictive validity of self-reported and digital trace measures is highly comparable, (b) a *d* between 0.50 and 0.79 indicates that the predictive validity of self-reported and digital trace measures is moderately comparable, and (c) a *d* higher than 0.80 indicates that the predictive validity of self-reported and digital trace measures is hardly comparable^25^.

#### Between-person comparative predictive validity

As Table 2 shows, while the self-reported measure of TSM was negatively and significantly associated with self-esteem (β = − 0.208, *p* = 0.008), the digital trace measure of TSM was negatively but not significantly associated with self-esteem (β = − 0.104, *p* = 0.116). This difference in the magnitude of the associations was not significant (*z* = − 1.26, *p* = 0.21). Likewise, the self-reported measure of TSM was negatively and significantly associated with well-being (β = − 0.242, *p* = 0.002), whereas the digital trace measure of TSM was negatively but not significantly associated with well-being (β = − 0.072, *p* = 0.202), a difference that was not significant (*z* = − 2.08, *p* = 0.04). Finally, both the self-reported (β = 0.049, *p* = 0.274) and digital trace measure of TSM (β = − 0.015, *p* = 0.433) were not related to friendship closeness, and these associations did not differ either (*z* = 0.76, *p* = 0.45). In all, on a between-person level, the predictive validity of self-reported and digital trace measures was highly comparable; both measures were non-significantly to weakly negatively associated with each indicator of psychosocial functioning and the magnitude of these associations did not differ significantly between methods.

**Table 2:** Between-person and within-person associations of time spent using social media (TSM) measured via self-reported and digital trace measures with self-esteem, well-being, and friendship closeness.

Component of psychosocial functioning	Self-report measure		Digital trace measure	
	β	*p*	β	*p*
**Self-Esteem**				
Between-person association	− .208	.008	− .104	.116
Within-person effect	− .030^a^	.009	− .009^b^	.222
**Well-Being**				
Between-person association	− .242	.002	− .072	.202
Within-person effect	.004^a^	.368	.058^b^	.000
**Friendship Closeness**				
Between-person association	.049	.274	− .015	.433
Within-person effect	.094	.000	.096	.000

βs are standardized using the STDYX Standardization in Mplus. *p*-values < .025 are significant. βs within rows that do not share the same superscript (^a,b^) are significantly different across self-reported and digital trace measures of TSM in a *t*-test (*p* < .017; corrected for multiple comparisons).

#### Within-person comparative predictive validity

Table 2 demonstrates that both the self-reported and digital trace measures of TSM yielded a very small negative within-person effect on self-esteem (β = − 0.030, *p* = 0.009 vs. β = − 0.009, *p* = 0.222), although this small difference was significant (*t*(158) = 2.85, *p* = 0.005, *d* = 0.23). As for well-being, we found no effect of self-reported TSM (β = 0.004, *p* = 0.368), but a small positive effect for the digital trace measure of TSM (β = 0.058, *p* < 0.001), a moderate difference in predictive validity between measures of TSM that was also significant (*t*(158) = 9.93, *p* < 0.001, *d* = 0.79). Finally, both the self-reported and digital trace measure had a significant positive within-person effect on friendship closeness (β = 0.094, *p* < 0.001 vs. β = 0.096, *p* < 0.001). These effects did not differ from each other (*t*(158) = 0.43, *p* = 0.67, *d* = 0.03). Overall, the within-person associations of TSM with the components of psychosocial functioning did not differ or only minimally (with β = 0.054) across measurement methods; the predictive validity of self-reported and digital trace measures of TSM was moderately to highly comparable.

**Figure 1:**
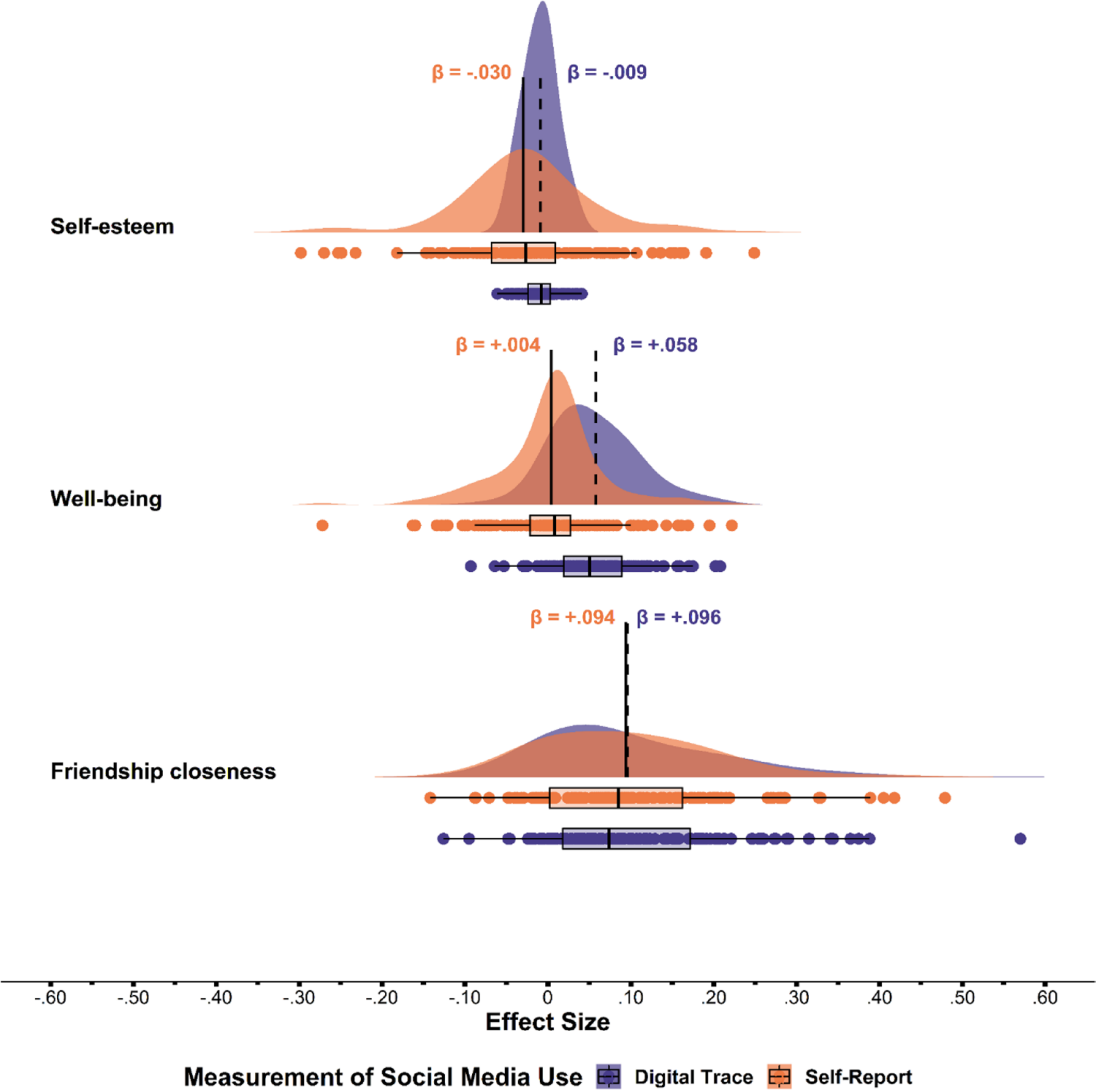
Distribution and overlap of the person-specific effects of time spent on social media (TSM) according to the self-reported and digital trace measure on self-esteem (top), well-being (middle), and friendship closeness (bottom). *Note.* Standardized effect sizes are displayed on the *x*-axis, the type of psychosocial functioning on the *y*-axis. The dots below the distribution plots represent the person-specific effects of each adolescent. The vertical solid and dashed black lines represent the overall within-person effects for the self-reported measure (solid) and the digital trace measure (dashed).

In addition to the overall within-person effects of TSM on each of the three components of psychosocial functioning, we examined the variance around these effects. Investigating this variance is important, because even when the overall within-person effects of the self-reported and digital trace measures of TSM correspond with each other, the variance around the effects might still differ. As the top part of Fig. 1 shows, the variances around the effect of the self-reported measure versus the digital trace measure of TSM on self-esteem differed. While only 2% of adolescents experienced changes in their self-esteem according to the digital trace measure of TSM (i.e., almost all effects clustered around zero), a substantial group experienced a positive (12%) or negative (33%) effect according to the self-reported TSM (i.e., a flatter distribution).

As the middle part of Fig. 1 shows, the distribution of the effects of the self-reported measure of TSM on well-being was comparable to that of the digital trace measure but it was more skewed to the right: More adolescents experienced a positive effect of TSM on well-being when measured via the digital trace measure (50%) than via the self-reported measure (15%). For friendship closeness (lower part of Fig. 1), the variances around the effects of the self-reported and digital trace measure were nearly identical (i.e., almost perfectly overlapping distributions).

#### Person-specific comparative predictive validity

Table 3 presents for what percentage of adolescents there was correspondence between their person-specific effect for the self-reported and the digital trace measure of TSM on self-esteem, well-being, and friendship closeness. As this table shows, the person-specific effects of the self-reported TSM corresponded with the person-specific effects of the digital trace measure of TSM among the majority of adolescents. Specifically, there was a 56% correspondence for self-esteem, 51% correspondence for well-being, and 78% correspondence for friendship closeness. These percentages imply that, for all three components of psychosocial functioning, the majority of the adolescents experienced an effect with the same direction (i.e., positive, negative, or null) for both the self-reported and digital trace measure of TSM.

**Table 3:** Overlap of Person-Specific Effects of the Self-Reported and Digital Trace Measure of Time Spent on Social Media (TSM) on Self-Esteem, Well-Being, and Friendship Closeness.

TSM	Digital trace ( +)	Digital trace (−)	Digital trace (0)
	*n* (%)	*n* (%)	*n* (%)
**Self-esteem**			
Self-Report ( +)	**0 (0)**	0 (0)	19 (12)
Self-Report (-)	0 (0)	**2 (1)**	50 (31)
Self-Report (0)	0 (0)	1 (1)	**87 (55)**
**Well-being**			
Self-Report ( +)	**20 (13)**	0 (0)	3 (2)
Self-Report (-)	9 (6)	**2 (1)**	14 (9)
Self-Report (0)	51 (32)	1 (1)	**59 (37)**
**Friendship closeness**			
Self-Report ( +)	**85 (53)**	0 (0)	16 (10)
Self-Report (-)	0 (0)	**0 (0)**	4 (3)
Self-Report (0)	13 (8)	2 (1)	**39 (25)**

*N* = 159. +  = positive effect; – = negative effect; 0 = no effect.

The **bold** *n*s and percentages on the diagonals indicate positive, negative, and no to very small effects of TSM that overlap across self-reported and digital trace measures. For example, as for self-esteem, 0 out of 159 adolescents experienced a positive effect of TSM according to both self-reported and digital trace measures, while 2 out of 159 adolescents (1%) experienced a negative effect, and 87 out of 159 adolescents (55%) a not to very small effect according to both the self-reported and digital trace measures of TSM Therefore, in total, the person-specific effects of self-reported and digital trace measures of TSM on self-esteem overlapped for 89 out of 159 adolescents (56%). As there are no guidelines to interpret the comparative predictive validity on a person-specific level, we focused on the overlap of the person-specific effects of the self-reported and digital trace measure of TSM on psychosocial functioning.

The percentual correspondence in the effects of self-reported and digital trace measures of TSM on each of the three components of psychosocial functioning was confirmed by the correlations among the person-specific effects of the self-reported and digital trace measures of TSM (*r*_self-esteem_ = 0.35; *r*_well-being_ = 0.37; *r*_friendship closeness_ = 0.64; see Supplementary Table S2 (https://osf.io/xzayk/) for the full correlation matrix). Following the guidelines by Gignac and Szodorai^26^, we interpreted that the person-specific effects were highly comparable as all correlations among the person-specific effects were above the threshold for a large correlation of *r* > 0.30. These findings indicate that adolescents who experienced more self-esteem, well-being, and friendship closeness due to more self-reported TSM, also displayed more self-esteem, well-being, and friendship closeness due to more TSM according to the digital trace measure. So, on a person-specific level, the predictive validity of the self-reported and the digital trace measure of TSM was highly comparable.

### Correspondence across three components of psychosocial functioning

The person-specific approach also allowed us to explore whether adolescents who experienced positive, negative, or null effects of TSM on one component of psychosocial functioning experienced similar effects on the two other components. There was a strong positive and significant association between the person-specific effects of TSM on self-esteem and well-being for both the self-reported and digital trace measures of TSM (*r*_self-report_ = 0.48; *r*_digital trace_ = 0.53). In addition, there was a positive and significant association between the person-specific effects of TSM on friendship closeness and well-being for both the self-reported and digital trace measures of TSM (*r*_self-report_ = 0.30; *r*_digital trace_ = 0.26), as well as between the effects on friendship closeness and self-esteem (*r*_self-report_ = 0.41; *r*_digital trace_ = 0.26). These findings suggest that adolescents who reported higher well-being after more TSM were also more likely to report higher self-esteem after more TSM, according to both the self-reported and digital trace measures. Similarly, adolescents who reported higher well-being or had more self-esteem after more TSM also felt closer to their friends after more TSM.

## Discussion

The aim of this study was to investigate the comparative predictive validity of self-reported and digital trace measures of time spent on social media (TSM) in a three-week experience sampling method (ESM) study among adolescents. Overall, we found that both self-reported and digital trace measures of TSM were weakly associated with psychosocial functioning, indicating that their predictive validity was highly comparable. Furthermore, our findings were consistent with earlier studies that found that self-reported measures of TSM lead to overestimating of actual TSM. Consistent with earlier studies ESM studies^13,23,27^ and a meta-analysis^11^, adolescents overestimated their TSM by a factor of two. In line with the meta-analysis of Parry et al.^11^, we also found poor convergent validity of self-reported measures of TSM. That is, the intercorrelations of self-reported and digital trace measures of TSM (*r* = 0.44) did not meet the threshold of *r* = 0.50 for minimum acceptable convergent validity^28^.

Based on our results and those reported in earlier studies, one could conclude that self-reported measures lead to inaccurate and invalid estimates of TSM and should be avoided in future studies into the effects of TSM. We do think, though, that it would be premature to dismiss self-reported measures of TSM altogether for three important reasons. First, inaccurate self-reported measures of TSM might be problematic if some adolescents overestimate their use while other adolescents do not. This would be especially problematic if third variables (e.g., gender or age) affected both the likelihood that adolescents overestimate their behavior as well as their scores on psychosocial functioning. In that case, the effects of self-reported estimates of TSM on psychosocial functioning would be biased. However, since almost all adolescents overestimated their social media use^23^, it is relatively unlikely that the inaccuracy affected our results. With regard to the media effects that occur within persons, we examined how deviations from adolescents’ own TSM covaried with their subsequent levels of psychosocial functioning across three weeks. We have no reason to assume that adolescents are more likely to overestimate their social media use at certain measurement occasions than at others. It is therefore unlikely that the overestimating of TSM by the large majority of adolescents affected the between-and within-person predictive validity of ESM self-reports^29^.

A second reason why we should not dismiss self-reported measures of TSM is that the insufficient convergent validity might also be caused by three shortcomings of digital trace measures of TSM. Although the overall validity of Android log data is generally high^30^, app crashes and bugs in either the tracking software or the individual smartphones of the participants could have caused the lower TSM obtained from digital trace measures. Furthermore, it is unknown and typically not checked whether adolescents are actually paying attention to their screen when their app usage is recorded. It is possible, for example, that social media apps are active on an adolescents’ smartphone while they, for example, watch television or talk to friends or family members (depending on how long the screen remains active without interaction from the user). Finally, digital trace measures typically record app usage on a single device (e.g., a smartphone). Yet, a considerable number of participants also access social media platforms through other devices or via web-based versions of the apps^23^, which might lower the accuracy of digital trace measures. This lower accuracy due to multiple-device or web-based social media use might be circumvented by the phrasing of the questions (e.g., by specifying the device used). As a result, the low correlations between self-reported and digital trace measures of TSM (i.e., convergent validity) might at least in part be due to a random underestimation of TSM caused by errors in digital trace measures rather than the more systematic overestimation caused by self-reported measures of TSM.

A third reason why we should not write off self-reported measures of TSM in future research is related to our previous point that digital trace measures might not be the gold standard against which self-reported measures of TSM should be judged. Our results clearly show that the convergent validity of the self-reported measure of TSM is not necessarily a requirement for the extent to which the predictive validity of ESM self-reports and digital trace measures are comparable, the type of validity that is crucial when investigating the effects of social media use. In fact, we found high comparability in the predictive validity of self-reported and digital trace measures of TSM for friendship closeness. Not only did the between-person association of self-reported TSM with friendship closeness strongly correspond with its digital trace equivalent (*r* = 0.049 vs. *r* = − 0.015), but also its within-person associations (β = 0.094 vs. β = 0.096). Moreover, as Fig. 1 shows, the distributions of the person-specific effects of self-reported and digital trace measures of TSM on friendship closeness that we explored were practically identical. In fact, 78% of adolescents experienced a similar (positive, negative, or null) effect for both the self-reported and digital trace measure of TSM.

Although the between-person associations of self-reported versus digital trace measures with self-esteem and well-being were not significantly different, the within-person and person-specific associations did differ significantly for both outcomes, although these differences could have been caused by the high power of within-person analyses based on 12,617 within-person assessments. After all, the differences in the within-person effects of self-reported versus digital trace estimates were small to moderate and the effects were in the same direction for both self-esteem (β = − 0.030 vs. β = − 0.009) and well-being (β = 0.004 vs. β = 0.058). Moreover, all effect sizes for the effects of self-reported measures and digital trace measures of TSM on self-esteem and well-being fell within the effect ranges reported in meta-analyses^5–7^. These findings imply that, even though we found that self-reported measures of TSM show poor *convergent validity*, we do find that their *predictive validity* is comparable to that of the digital trace measure.

Even though all associations of TSM with psychosocial functioning were small to moderate and in line with those reported in earlier studies, we found somewhat less overlap in the person-specific effects of self-reported and digital trace measures of TSM on self-esteem and well-being than on friendship closeness. It is unlikely that TSM is the cause of these discrepancies since we used the same self-reported measure of TSM for all three outcome variables. Moreover, if digital trace measures would have less measurement error than self-reported measures, the patterns would have been consistent across all outcome variables. It is more plausible that differences in the associations are caused by differences in the outcome variables. Whereas friendship closeness taps into the social component of psychosocial functioning, both self-esteem and well-being cover the personal component of psychosocial functioning^1^. Since social media are inherently social (e.g., to maintain relationships with existing friends), time spent on these media might more easily lead to consistent (mostly positive) effects of self-reported and digital trace measures of TSM on friendship closeness. In contrast, as has been shown by Valkenburg et al.^17^, the personal components self-esteem and well-being might be more responsive to the valence (i.e., positivity or negativity) of social media interactions than to general time spent on social media. As a result, both self-reported and digital trace measures of TSM might be suboptimal to fully understand the effects of social media use on self-esteem and well-being, which might explain the somewhat greater discrepancies in the person-specific effects on these outcomes.

The current study provided a renewed look on the validity of self-reported measures of TSM. The focus of this study was on the validity of ESM self-reports and not of retrospective self-reports (e.g., how much time did you spend on Instagram in the previous week?). An important distinction between ESM self-reports and retrospective self-reports is that ESM self-reports are less subject to recall bias than retrospective self-reports^31^. But although the convergent validity of ESM self-reports is not superior to that of retrospective self-reports^23^, it is still largely unknown whether the predictive validity of ESM and retrospective self-reports is comparable. Nevertheless, both self-reported and digital trace measures of TSM are just what they are: measures of time spent on social media. Investigating TSM might be too coarse to yield valid effects on outcomes like self-esteem and well-being^32^. To obtain a true understanding of social media use and its effects, future research should pay more attention to the content of adolescents’ social media use and employ both self-reported and digital trace measures that are able to tap into the content of social media use.

## Method

This preregistered study is part of an intensive longitudinal cohort study (https://osf.io/327cx) that investigates the effect of adolescents’ TSM on their psychosocial functioning. The study was approved by the Ethics Review Board of the Faculty of Social and Behavioral Sciences at the University of Amsterdam and was performed in accordance with the guidelines formulated by the Ethics Review Board. The study consists of two three-week ESM studies fielded in December 2019 and June 2020, as well as 16 biweekly retrospective surveys. The current study uses data belonging to the second ESM study and extends the study by Verbeij et al.^23^, which was based on the same ESM wave and which also tracked app usage and screen state data (the digital trace measure) of adolescents with an Android smartphone. A detailed timeline of the larger project can be found on OSF (https://osf.io/fb945). The second ESM wave started on 3 June 2020, which coincidentally happened to be the day that the mandated school closures due to COVID-19 in the Netherlands ended after 2.5 months.

### Participants

We recruited adolescents through a secondary school in the south of the Netherlands. At the start of the larger project, researchers informed the school, parents, and the adolescents of the aim and procedure of the study. Both parents and adolescents were informed that adolescents’ responses would be treated confidentially. We obtained informed assent from all adolescents and informed consent from a parent or legal guardian. Since the tracking software could only track Android smartphones, the potential sample consisted of 171 adolescents. Out of these 171 adolescents, 159 (92%) provided active consent to track their app usage. The final sample of this study therefore consisted of a subsample of 159 middle adolescents (*M*_*age*_ = 14.0 years, *SD*_*age*_ = 0.69, 47% girls), of whom 98% identified themselves as Dutch. The educational levels of the sample were representative of the specific region in the Netherlands: 40.3% were enrolled in the prevocational secondary education track, 33.3% in the intermediate general secondary education track, and 26.4% in the academic preparatory education track.

### Procedure

Adolescents received online instructions about how to install the ESM application *Ethica*^33^ on their own smartphone. Additionally, they were asked to install the *Ethica App Usage Stream*^19^ application on their smartphone, which tracked their app usage (i.e., type of app and duration of use) during the three-week study period. In addition to tracking adolescents’ app usage, we also collected screen state data through the *Ethica* app. This allowed us to check if (and at what time) adolescents’ smartphone screens were turned on or off.

Adolescents were asked, through the *Ethica* app, to fill in a pre-ESM self-report to indicate which social media platforms they used *more* than once per week (i.e., Instagram [131 adolescents], WhatsApp [156 adolescents], and Snapchat [108 adolescents]). If they indicated that they used a platform more than once per week, we asked them to report on their use of that platform in all subsequent ESM self-reports. If adolescents used any of these platforms less frequently, we asked questions about other platforms (i.e., YouTube, gaming) or activities so that each adolescent received the same number of questions in the ESM study.

The ESM study started two weeks after completion of the pre-ESM self-report. The *Ethica* app installed on adolescents’ smartphones was programmed to generate six notifications per day for a period of three weeks (i.e., a total of 126 ESM self-reports; see our notification scheme [https://osf.io/tbdjq] for more information). Each self-report took about two minutes to complete. Adolescents received €0,30 for completing an ESM self-report and €0,50 for completing the final ESM self-report of the day. At the start of each day, adolescents who completed all six self-reports on the previous day were entered into a lottery, in which four adolescents could win €25. A total of 20,034 ESM self-reports were sent, but six were not received by adolescents due to technical errors. Of the 20,028 self-reports received, adolescents (partially) completed 12,617 self-reports (net compliance of *M* = 63%, min = 3%, max = 100%). On average, adolescents completed 79.4 ESM self-reports (*SD* = 34.8; range 4–126; median = 92). Further information about the participant recruitment and compliance for the overall project can be found in the sample and compliance overview (https://osf.io/8f7ds).

### Measures of time spent with social media

#### Self-reported ESM measure

In each ESM survey, adolescents were asked to indicate their response to the question “In the previous hour, how much time did you use [Instagram/WhatsApp/Snapchat]?” using response options ranging from 0 to 60 min on a horizontal slider, with 1-min intervals.

#### Digital trace measure

Adolescents’ time spent on Instagram, WhatsApp, and Snapchat was tracked continuously during the three-week ESM period by the *Ethica App Usage Stream* application. Every five minutes, this application retrieved the Android log data that were stored on adolescents’ smartphone. These log data recorded the foreground time of all applications, including Instagram, WhatsApp, and Snapchat, which can be defined as time spent on the applications when the adolescents’ smartphone was unlocked. To control for the possibility that adolescents’ smartphones still recorded app usage when apps were running in the background while their smartphone screen was turned off, we excluded records of app use when adolescents’ screen was turned off (based on the screen state data; 2.6% of the data of the digital trace measure was excluded). Per social media platform, we determined TSM in the hour before each ESM self-report according to the digital trace measure. The digital trace measure was cleaned based on the procedure of Verbeij et al.^23^ (also see https://osf.io/jkre2/).

### Measures of psychosocial functioning

We measured three components of adolescents’ psychosocial functioning: self-esteem, well-being, and friendship closeness. All items were pre-tested in a pilot study (https://osf.io/nhks2/).

#### Self-esteem

In line with studies that established the validity of single-item measures of self-esteem^34^, in every self-report survey, adolescents were asked to indicate their response to the question “How satisfied do you feel about yourself right now?” using a 6-point scale ranging from 0 (*not at all*) to 6 (*completely*), with 3 (*a little*) as the midpoint. The validity of this single-item measure of self-esteem has been established^34^.

#### Well-being

In every self-report survey, adolescents were asked to respond to the question “How happy do you feel right now?” using a 6-point scale ranging from 0 (*not at all*) to 6 (*completely*), with 3 (*a little*) as the midpoint. This single-item instrument has been reliably used in previous ESM studies^15^ and has high convergent validity as indicated by strong between- and within-person associations with positive and negative affect^15^.

#### Friendship closeness

In line with previous studies^35^, in every self-report survey, adolescents were asked to answer the question “How close to your friends do you feel right now?” using a 6-point scale ranging from 0 (*not at all*) to 6 (*completely*), with 3 (*a little*) as the midpoint. We specifically focused on close friends to ensure that adolescents did not consider all their social media connections as friends. Adolescents in this sample defined close friends as supportive, responsive, and accessible peer relationships, which is in line with the definition of friendship^22^ and with the social provisions that characterize friendship^36^.

### Statistical analyses

Unless stated otherwise, we exactly followed our preregistered analysis plan (https://osf.io/krs8j). To create indices of total TSM, we summed the ESM self-reports as well as the digital trace measure across the three social media platforms. Sum scores exceeding 60 min were recoded to 60 min. To make the scales of TSM and the different psychosocial functioning measures comparable, we divided the TSM variables (0–60 min) by ten. This resulted in a continuous scale running from 0 to 6, so that an increase of TSM with 1 unit reflects an increase of 10 min. We used these sum scores as predictors of the three psychosocial functioning components in our analyses.

We checked the assumption of stationarity according to our pre-registered analysis plan. The data met the stationarity assumption, as only 0%, 0.3%, and 0.1% of the variance in self-esteem, well-being, and friendship closeness was explained by day of the study. These findings indicate that adolescents’ levels of self-esteem, well-being, and friendship closeness did not change over the course of the three-week study.

To investigate the between-person associations, within-person effects, and person-specific effects of the self-report and digital trace measure of TSM on psychosocial functioning, we estimated six autoregressive lag-1 models (AR[1] models) using dynamic structural equation modeling (DSEM) in Mplus 8.5. For each of the three outcome measures (i.e., self-esteem, well-being, and friendship closeness), we estimated a model with the self-reported measures of TSM and a model with the digital trace measure of TSM as the predictor (i.e., time-varying covariate). Following the procedure of McNeish and Hamaker^37^, we used the self-reported and digital trace measure of TSM measured at the same time point as adolescents’ psychosocial functioning. Since these measurements refer to different time spans (i.e., “the past hour” for time spent on social media versus “right now” for psychosocial functioning), temporal precedence, a condition for causality^38^, is implied by the different time span references of the measurements.

Each model was split into two levels: the within-person and between-person level. At the within-person level, we specified the self-reported and digital trace measure of TSM as the time-varying covariates of each respective component of psychosocial functioning. We also controlled for the autoregressive effect of each component of psychosocial functioning (e.g., well-being predicted by lag-1 well-being). At the between-person level, we included the respective latent mean levels of adolescents’ psychosocial functioning (i.e., self-esteem, well-being, and friendship closeness), the latent mean of the self-reported and digital trace measure of TSM, the mean autoregressive effect of psychosocial functioning, and the correlation between these mean levels. We also estimated the between-person variance around the within-person effects of TSM on self-esteem, well-being, and friendship closeness (i.e., random effects). Using this two-level approach, we could obtain person-specific effects of TSM on each component of psychosocial functioning (i.e., one effect per adolescent), the overall within-person effect (i.e., the average of the person-specific effects), and the variance around the overall within-person effect.

We assessed the comparative predictive validity by investigating correspondence between the associations of the self-reported and digital trace measure of TSM with self-esteem, well-being, and friendship closeness. We assessed this correspondence on a between-person, within-person, and person-specific level. On the between-person level, we investigated the correspondence of the *between-person associations* of the self-reported and digital trace measure of TSM with well-being, self-esteem, and friendship closeness. The interpretation of between-person associations was based on Gignac and Szodorai^26^. Associations ranging from − 0.10 to + 0.10 were interpreted as “non-existent to very small” and all associations beyond this range as negative or positive. To test the correspondence of the *between-person* associations of the self-reported and digital trace measure of TSM with adolescents’ psychosocial functioning, we used the “*cocor.dep.groups.overlap*” of the package Cocor in *R*^39^. This resulted in three *z*-tests, one for each aspect of psychosocial functioning (i.e., self-esteem, well-being, and friendship closeness). For these *z*-tests we adjusted our alpha-level based on the number of tests we performed (α = 0.05/3 = 0.017).

We also assessed the correspondence of the *within-person effects* of the self-reported and digital trace measure of TSM on well-being, self-esteem, and friendship closeness. We used raincloud plots^40^ (i.e., a combination of density plots, boxplots, and scatter plots) to visualize the overlap across the within-person effects of the self-reported and digital trace measure of TSM on self-esteem, well-being, and friendship closeness (i.e., overlap of the overall within-person effect and the variance around the overall within-person effect). Following Adachi and Willoughby^41^ and Meier and Reinecke^42^, we interpreted effects ranging from -0.05 to + 0.05 as “non-existent to very small”, and all effect sizes beyond this range as “negative” or “positive,” respectively. We tested per outcome whether the overall *within-person* effect of the self-reported and digital trace measure of TSM differed, using the “*t.test*” function of the package Stats in *R.* This resulted in three paired *t*-tests, one for each aspect of psychosocial functioning (i.e., self-esteem, well-being, and friendship closeness). For these *t*-tests we adjusted the alpha-level based on the number of tests we performed (α = 0.05/3 = 0.017). We also calculated Cohen’s *d* to compare the magnitude of the difference in the *within-person* effects between the self-reported and digital trace measure of TSM on adolescents’ psychosocial functioning. Since our method to determine the comparative predictive validity is novel, we followed the general guidelines for interpreting Cohen’s *d*, where (a) a *d* between 0.20 and 0.49 indicates a small difference, (b) a *d* between 0.50 and 0.79 indicates a moderate difference, and (c) a *d* higher than 0.80 indicates a large difference^25^.

Finally, we investigated the correspondence of the *person-specific effects* with regard to the two different measures of TSM (self-reported measure vs. digital trace measure) and we also explored the person-specific correspondence for three different outcome measures (i.e., self-esteem vs. well-being vs. friendship closeness). We investigated the person-specific correspondence for the self-reported and digital trace measure of TSM, by computing the percentual overlap in person-specific effects. We also calculated, per outcome, the correlation between the person-specific effects of TSM according to the self-reported measure versus the digital trace measure. In addition, we explored the correlations between the person-specific effects of TSM on self-esteem, well-being, and friendship closeness for the self-reported and digital trace measure separately. To shed light on the magnitude of the person-specific comparative predictive validity, we interpreted the correlations according to the guidelines of Gignac and Szodorai^26^. A correlation ranging from (a) -0.10 < *r* < 0.10 was interpreted as “non-existent to very small” (i.e., non-comparable), (b) 0.10 ≤ *r* < 0.20 was interpreted as “small” (i.e., lowly comparable), (c) 0.20 ≤ *r* < 0.30 was interpreted as “moderate” (i.e., moderately comparable), and (d) *r* ≥ 0.30 was interpreted as “large” (i.e., highly comparable).

We estimated the models with a minimum number of 5,000 iterations. Due to convergence issues, we deviated from our preregistered analysis plan by changing the time interval from 2 to 3 h (TINTERVAL = 3) and by estimating six instead of two separate autoregressive lag-1 models (AR[1] models). That is, a model with the self-reported measure and a model with the digital trace measure for each component of psychosocial functioning separately. Our six final models converged successfully as the Potential Scale Reduction (PSR) values were very close to 1 (Gelman & Rubin, 1992). Since the parameter trace plots contained less trends and spikes when we doubled the number of iterations, we reported the results of the models with a minimum of 10,000 iterations. The main results of our models are reported in Supplementary Table S1(https://osf.io/kq5yg/); in the results section we report the results concerning our research questions.

#### Sensitivity analyses

We performed three preregistered sensitivity analyses to test for the robustness of our results. In these sensitivity analyses, we (a) included all adolescents in the models that were based on the self-reported measure rather than only those with data on the digital trace measure, (b) included adolescents who only used social media on their smartphone, (c) excluded adolescents with potentially untrustworthy response patterns. Overall, the results of these different sensitivity analyses did not deviate substantially from our default models. See Supplementary Table S3 (https://osf.io/exzgr/) for a detailed overview of the results of these sensitivity analyses.

## Supplementary Information

Supplementary Information.

## Data Availability

The preregistration of the hypotheses, design, sampling and analysis plan, and the analysis scripts used for this paper are available online on OSF (https://osf.io/4gaqk/). The anonymous dataset is published on Figshare (https://doi.org/10.21942/uva.16780204).
