# Supplementary material for: Experience sampling self-reports of social media use have comparable predictive validity to digital trace measures
Source: Sci Rep. 2022 May 9;12:7611. doi: 10.1038/s41598-022-11510-3 (PMC9084269; doi:10.1038/s41598-022-11510-3)
Supplement: Supplementary file 1 — Supplementary Information. [file 41598_2022_11510_MOESM1_ESM.docx]

Supplementary Table S1

| *Full Overview of DSEM Models: Between- and Within-Person Associations of Time Spent Using Social Media (TSM) on the Self-Reported and Digital Trace Measure, With Self-Esteem (SE), Well-Being (WB), and Friendship Closeness (FCL)* | | | | | | | | | |
| --- | --- | --- | --- | --- | --- | --- | --- | --- | --- |
|  | Self-Reported Measure | | | |  | Digital Trace Measure | | | |
|  | *B* | β | *p* | 95% CI |  | *B* | β | *p* | 95% CI |
| Between-Person |  |  |  |  |  |  |  |  |  |
| TSM & SE | -0.253 | -.208 | .008 | [-.358, -.045] |  | -0.049 | -.104 | .116 | [-.269, .065] |
| TSM & WB | -0.311 | -.242 | .002 | [-.391, -.084] |  | -0.036 | -.072 | .202 | [-.238, .096] |
| TSM & FCL | 0.072 | .049 | .274 | [-.113, .210] |  | -0.009 | -.015 | .433 | [-.183, .149] |
| Within-Person Effects |  |  |  |  |  |  |  |  |  |
| TSM 🡪 SE | -0.030 | -.030 | .009 | [-.054, -.006] |  | -0.009 | -.009 | .222 | [-.030, .013] |
| TSM 🡪 WB | 0.004 | .004 | .368 | [-.022, .028] |  | 0.069 | .058 | .000 | [.033, .082] |
| TSM 🡪 FCL | 0.124 | .094 | .000 | [.070, .118] |  | 0.163 | .096 | .000 | [.070, .121] |
| SE (t-1) 🡪 SE (t) | 0.188 | .188 | .000 | [.161, .214] |  | 0.190 | .190 | .000 | [.162, .218] |
| WB (t-1) 🡪 WB (t) | 0.180 | .180 | .000 | [.155, .206] |  | 0.196 | .197 | .000 | [.169, .225] |
| FCL (t-1) 🡪 FCL (t) | 0.223 | .223 | .000 | [.197, .248] |  | 0.233 | .233 | .000 | [.207, .257] |
|  | σ^2^ | *p* |  | 95% CI |  | σ^2^ | *p* |  | 95% CI |
| Random Effects |  |  |  |  |  |  |  |  |  |
| TSM 🡪 SE^a^ | 0.018 | .000 |  | [0.010, 0.028] |  | 0.004 | .000 |  | [0.001, 0.011] |
| TSM 🡪 WB^a^ | 0.012 | .000 |  | [0.007, 0.021] |  | 0.013 | .000 |  | [0.006, 0.025] |
| TSM 🡪 FCL^a^ | 0.040 | .000 |  | [0.027, 0.060] |  | 0.070 | .000 |  | [0.045, 0.108] |
| SE (t-1) 🡪 SE (t) | 0.040 | .000 |  | [0.028, 0.058] |  | 0.043 | .000 |  | [0.030, 0.061] |
| WB (t-1) 🡪 WB (t) | 0.044 | .000 |  | [0.031, 0.061] |  | 0.038 | .000 |  | [0.026, 0.055] |
| FCL (t-1) 🡪 FCL (t) | 0.039 | .000 |  | [0.027, 0.060] |  | 0.039 | .000 |  | [0.028, 0.054] |
| Other Variances |  |  |  |  |  |  |  |  |  |
| SE (within-person) | 0.908 | .000 |  | [0.885, 0.931] |  | 0.919 | .000 |  | [0.897, 0.943] |
| SE (between-person) | 1.076 | .000 |  | [0.858, 1.371] |  | 1.075 | .000 |  | [0.860, 1.370] |
| WB (within-person) | 0.893 | .000 |  | [0.871, 0.917] |  | 0.896 | .000 |  | [0.874, 0.919] |
| WB (between-person) | 1.180 | .000 |  | [0.942, 1.508] |  | 1.184 | .000 |  | [0.949, 1.508] |
| FCL (within-person) | 1.551 | .000 |  | [1.512, 1.590] |  | 1.547 | .000 |  | [1.507, 1.587] |
| FCL (between-person) | 1.521 | .000 |  | [1.210, 1.928] |  | 1.528 | .000 |  | [1.215, 1.957] |

*Note.* βs are standardized using the STDYX Standardization in Mplus. ^a^The random effect of time spent on social media on psychosocial functioning reflects the between-person variance around the within-person effect of the self-reported measure (Model 1) or the digital trace measure (Model 2) on well-being, self-esteem, and friendship closeness respectively. *p*-values < .025 are significant.

Supplementary Table S2

*Correlation Matrix for the Person-Specific Effects: The Person-Specific Effects of Time Spent Using Social Media (TSM) on the Self-Reported and Digital Trace Measure on Self-Esteem, Well-Being, and Friendship Closeness*

|  | 1 | 2 | 3 | 4 | 5 | 6 |
| --- | --- | --- | --- | --- | --- | --- |
| 1. TSM (SR) 🡪 Self-Esteem | — | — | — | — | — | — |
| 1. TSM (SR) 🡪 Well-Being | 0.48^***^ | — | — | — | — | — |
| 1. TSM (SR) 🡪 Friendship Closeness | 0.41^***^ | 0.30^***^ | — | — | — | — |
| 1. TSM (DT) 🡪 Self-Esteem | 0.35^***^ | 0.15 | 0.28^***^ | — | — | — |
| 1. TSM (DT) 🡪 Well-Being | 0.24^**^ | 0.37^***^ | 0.19^*^ | 0.53^***^ | — | — |
| 1. TSM (DT) 🡪 Friendship Closeness | 0.16^*^ | 0.05 | 0.64^***^ | 0.26^***^ | 0.26^**^ | — |

*Note*. TSM = Time spent on social media in the previous hour. SR = Self-reported measure.
DT = Digital trace measure. ^*^*p* < .05. ^**^*p* < .01. ^***^*p* < .001.

Supplementary Table S3a

| \| *Sensitivity Analysis: Between-Person and Within-Person Associations of the Time Spent Using Social Media (TSM) measured via Self-Reported with Self-esteem, Well-being, and Friendship Closeness, in All Adolescents (N = 300)* \| \| \| \| \| \| \| --- \| --- \| --- \| --- \| --- \| --- \| \|  \| Main Models  *N* = 159 \| \|  \| All Adolescents  *N* = 300 \| \| \|  \| β \| *p* \|  \| β \| *p* \| \| ***Between-Person Associations*** \|  \|  \|  \|  \|  \| \| Time Spent on Social Media & Self-Esteem \| -0.208 \| .008 \|  \| -0.138 \| .012 \| \| Time Spent on Social Media & Well-Being \| -0.242 \| .002 \|  \| -0.161 \| .002 \| \| Time Spent on Social Media & Friendship Closeness \| 0.049 \| .274 \|  \| 0.065 \| .149 \| \| ***Within-Person Effects*** \|  \|  \|  \|  \|  \| \| Time Spent on Social Media 🡪 Self-Esteem \| -0.030 \| .009 \|  \| -0.012 \| .105 \| \| Time Spent on Social Media 🡪 Well-Being \| 0.004 \| .368 \|  \| 0.015 \| .038 \| \| Time Spent on Social Media 🡪 Friendship Closeness \| 0.094 \| .000 \|  \| 0.086 \| .000 \| \| *Note.* βs are standardized using the STDYX Standardization in Mplus. *p*-values below < .025 are significant.  Conclusion: After including all adolescents (N = 300), rather than only those with data on the digital trace measure (N = 159), the very small negative within-person effect of time spent on social media according to the self-reported measure on self-esteem was no longer significant (marked red). All other between-person associations and within-person effects were replicated. \| \| \| \| \| \| |
| --- | --- | --- | --- | --- | --- | --- | --- | --- | --- | --- | --- | --- | --- | --- | --- | --- | --- | --- | --- | --- | --- | --- | --- | --- | --- | --- | --- | --- | --- | --- | --- | --- | --- | --- | --- | --- | --- | --- | --- | --- | --- | --- | --- | --- | --- | --- | --- | --- | --- | --- | --- | --- | --- | --- | --- | --- | --- | --- | --- | --- | --- | --- | --- | --- | --- | --- | --- | --- | --- | --- | --- | --- |

Supplementary Table S3b

| *Sensitivity Analysis: Between-Person and Within-Person Associations of the Time Spent Using Social Media (TSM) on the Self-Reported and Digital Trace Measure With Self-Esteem, Well-Being, and Friendship Closeness, Among a Subsample of Adolescents who use Social Media only on their Smartphone (N = 122)* | | | | | | |
| --- | --- | --- | --- | --- | --- | --- |
|  |  | Self-Reported Measure | | | |  |
|  | Main Models (*N* = 159) | |  | Phone Only (*N* = 122) | | |
|  | β | *p* | β | | | *p* |
| ***Between-Person Associations*** |  |  |  |  | |  |
| Time Spent on Social Media & Self-Esteem | -0.208 | .008 |  | -0.162 | | .041 |
| Time Spent on Social Media & Well-Being | -0.242 | .002 |  | -0.196 | | .020 |
| Time Spent on Social Media & Friendship Closeness | 0.049 | .274 |  | 0.092 | | .172 |
| ***Within-Person Effects*** |  |  |  |  | |  |
| Time Spent on Social Media 🡪 Self-Esteem | -0.030 | .009 |  | -0.036 | | .004 |
| Time Spent on Social Media 🡪 Well-Being | 0.004 | .368 |  | 0.012 | | .197 |
| Time Spent on Social Media 🡪 Friendship Closeness | 0.094 | .000 |  | 0.102 | | .000 |
|  |  | Digital Trace Measure | | |  | |
|  | Main Model (*N* = 159) | |  | Phone Only (*N* = 122) | | |
|  | β | *p* |  | β | | *p* |
| ***Between-Person Associations*** |  |  |  |  | |  |
| Time Spent on Social Media & Self-Esteem | -0.104 | .116 |  | -0.050 | | .311 |
| Time Spent on Social Media & Well-Being | -0.072 | .202 |  | -0.048 | | .313 |
| Time Spent on Social Media & Friendship Closeness | -0.015 | .433 |  | 0.089 | | .186 |
| ***Within-Person Effects*** |  |  |  |  | |  |
| Time Spent on Social Media 🡪 Self-Esteem | -0.009 | .222 |  | -0.017 | | .135 |
| Time Spent on Social Media 🡪 Well-Being | 0.058 | .000 |  | 0.071 | | .000 |
| Time Spent on Social Media 🡪 Friendship Closeness | 0.096 | .000 |  | 0.096 | | .000 |
| *Note.* βs are standardized using the STDYX Standardization in Mplus. *p*-values below < .025 are significant. | | | | | | |
| Conclusion: After including adolescents who only use social media on their smartphone (N = 122), the between-person associations of time spent on social media according to the self-reported measure and self-esteem was no longer significant (marked red). All other between-person associations and within-person effects were replicated. | | | | | | |

Supplementary Table S3c

| *Sensitivity Analysis: Between-Person and Within-Person Associations of the Time Spent Using Social Media (TSM) measured via the Self-Reported Measure With Self-Esteem, Well-Being, and Friendship Closeness, Among a Subsample of Adolescents who Provided Potentially Untrustworthy Responses (N = 157)* | | | | | |
| --- | --- | --- | --- | --- | --- |
|  | Self-Reported Measure | | | | |
|  | Main Models (*N* = 159) | |  | Untrustworthy (*N* = 157) | |
|  | β | *p* |  | β | *p* |
| ***Between-Person Associations*** |  |  |  |  |  |
| Time Spent on Social Media & Self-Esteem | -0.208 | .008 |  | -0.198 | .010 |
| Time Spent on Social Media & Well-Being | -0.242 | .002 |  | -0.240 | .002 |
| Time Spent on Social Media & Friendship Closeness | 0.049 | .274 |  | 0.062 | .229 |
| ***Within-Person Effects*** |  |  |  |  |  |
| Time Spent on Social Media 🡪 Self-Esteem | -0.030 | .009 |  | -0.030 | .011 |
| Time Spent on Social Media 🡪 Well-Being | 0.004 | .368 |  | 0.006 | .322 |
| Time Spent on Social Media 🡪 Friendship Closeness | 0.094 | .000 |  | 0.096 | .000 |
| *Note.* βs are standardized using the STDYX Standardization in Mplus. *p*-values below < .025 are significant.  *Potentially untrustworthy responses*  1. *Within-person response consistency*: within-person correlation of at least *r* = +.10 between well-being, self-esteem, or friendship closeness (145 adolescents violated this check).  2. *No outliers*: person mean scores for time spent on social media or psychosocial functioning are not +/- 2 *SD* above/below person’s mean score (Self-reported measure: two adolescents violated this check; Digital trace measure: no adolescents violated this check).  3. *Open question check*: we checked adolescents’ responses to the open question of the self-reported measure of each day. We screened for unserious responses (1 adolescents violated this check).  If adolescents violated **at least two validation checks** above, we considered their answers as potentially untrustworthy:  Self-reported measure: 2 adolescents excluded (N = 157)  Digital trace measure: no adolescents excluded (N = 159; same as main model)  Conclusion: After excluding adolescents who provided potentially untrustworthy responses, all between-person associations and within-person effects were replicated. | | | | | |
